# An online tool for measuring and visualizing phenotype similarities using HPO

**DOI:** 10.1186/s12864-018-4927-z

**Published:** 2018-08-13

**Authors:** Jiajie Peng, Hansheng Xue, Weiwei Hui, Junya Lu, Bolin Chen, Qinghua Jiang, Xuequn Shang, Yadong Wang

**Affiliations:** 1 School of Computer Science, Northwestern Polytechnical University, Xi’an, 710072 China; 2 Department of Computer Science and Technology, Harbin Institute of Technology, Shenzhen, 518055 China; 3 School of Life Science and Technology, Harbin Institute of Technology, Harbin, 150001 China; 4 School of Computer Science and Technology, Harbin Institute of Technology, Harbin, 150001 China

**Keywords:** Human phenotype ontology, Web tool, Phenotype similarity

## Abstract

**Background:**

The Human Phenotype Ontology (HPO) is one of the most popular bioinformatics resources. Recently, HPO-based phenotype semantic similarity has been effectively applied to model patient phenotype data. However, the existing tools are revised based on the Gene Ontology (GO)-based term similarity. The design of the models are not optimized for the unique features of HPO. In addition, existing tools only allow HPO terms as input and only provide pure text-based outputs.

**Results:**

We present *PhenoSimWeb*, a web application that allows researchers to measure HPO-based phenotype semantic similarities using four approaches borrowed from GO-based similarity measurements. Besides, we provide a approach considering the unique properties of HPO. And, *PhenoSimWeb* allows text that describes phenotypes as input, since clinical phenotype data is always in text. *PhenoSimWeb* also provides a graphic visualization interface to visualize the resulting phenotype network.

**Conclusions:**

*PhenoSimWeb* is an easy-to-use and functional online application. Researchers can use it to calculate phenotype similarity conveniently, predict phenotype associated genes or diseases, and visualize the network of phenotype interactions. *PhenoSimWeb* is available at http://120.77.47.2:8080.

## Background

Since the successful completion of the Human Genome Project, significant improvement has been made in genome sequencing technologies, which benefit the Mendelian disease and cancer diagnosis [1–9]. Even so, it remains challenging to make correct diagnosis only based on sequencing technologies for many diseases. Because the relationships between genetic variants and clinical phenotypes are difficult to understand for diseases with high genetic heterogeneity and complex phenotypes [10, 11].

Patient phenotypes are the observable features of a patient, such as anatomy and biomedical properties [12]. Phenotypes are usually determined by both genetic and environmental factor. To improve the efficiency of disease diagnosis, several methods have been developed to analyse the relationships between patient phenotypes and known phenotypes related with a gene based on Human Phenotype Ontology (HPO) recently [13–15]. The Human Phenotype Ontology (HPO) is one of the most popular bioinformatics resources, which was constructed by Robinson et al. in 2008 [12]. HPO provides the unique and structured vocabulary to represent the phenotypic characteristics and their relationships with a directed acyclic graph (DAG). In recent study, quantifying the phenotypic similarity based on HPO is usually integrated with sequencing technologies to aid disease diagnosis [16–20].

As a kind of widely used resource, HPO contains abundant information and reasearchers could study phenotype semantic similarity conveniently. In recent years, various methods have been proposed to compute HPO-based phenotype similarities by comparing HPO terms with their annotations and topological information, such as Phenomizer [21], OWLSim [22] and HPOSim [23]. However, Most of these methods are modified based on GO-based similarity measurements that have been widely utilized and studied by many researchers [24–31]. Phenomizer applied information content to compute the phenotype semantic similarity based on HPO. Based on the IC-based method, PhenomeNet [32] and OWLSim [22] exploit simGIC [33] to measure the semantic similarity of two phenotype sets. HPOSim [23] implements seven commonly used ontology-based semantic similarity measurements to compute the phenotype similarities, such as Jiang measure [34], Schlicker measure [35] and Wang measure [31].

Although the aforesaid methods have been widely used to measure the phenotype similarity, none of these measurements take into account the unique features of HPO. To fill this gap, we have recently presented a measurement named PhenoSim to compute the phenotype similarities [36, 37] considering the unique properties of HPO. Our method can simulate the noise in the patient phenotype dataset and compute the similairties using a novel path-constrained Information Content based measurement in three steps. Firstly, construct a phenotype network. Secondly, reduce noise in the patient’s phenotype set using PageRank [38] algorithm. Thirdly, compute phenotype set similarities using a novel path-contrained Information Content based measurement. And the experiment result shows that PhenoSim performs better than existing methods.

In addition, existing tools have two main drawbacks: firstly, none of existing tools allow text that describes phenotype features as input, neglecting that symptoms of patients are always described as text not HPO terms; secondly, most existing tools ignore the effect of visualization, which is necessary for result interpreation, and simply list the experimental results as the final output. Thereby, it is very urgent and essential to research an easy-to-use and functional web applicaiton.

In this article, we present a novel and easy-to-use online application, termed as *PhenoSimWeb*, to compute phenotype similarities based on HPO and to visualize the similarity using an intuitive graphical interface. Comparing with the existing online tools, the main contributions of our work can be summarized as:

- *PhenoSimWeb* supplies researchers with a measurement based on the design optimized for unique features of HPO.
- *PhenoSimWeb* allows text that describes phenotype features as input.
- *PhenoSimWeb* contains an intuitive and functional visualization interface to visualize phenotype association network.

## Methods

*PhenoSimWeb* is a Browser/Server architecture-based online application which can be used to calculate the phenotype similarities based on HPO, visualize the association between phenotypes, and predict the associated gene/diseases given a set of phenotypes. The back-end of *PhenoSimWeb* is implemented using Java SDK 7, Python 2.7 and web framework web.py. And *PhenoSimWeb* uses MySQL to manage dataset. In part of data transmission between the browser and server, the web application applys JavaScript Object Notation (JSON) and Asynchronous JavaScript and XML (AJAX) and so on. Besides, *PhenoSimWeb* uses cytoscape.js and HTML5 canvas as the graphics engine for the association network visualization. The Human Phenotype Ontology (HPO) dataset was downloaded from the HPO official website (http://humanphenotype-ontology.github.io/) on January, 2016. *PhenoSimWeb* was tested on Chrome, Firefox and Internet Explorer.

## Results and discussion

*PhenoSimWeb* mainly contains two operations to execute: 1)to type in a set of phenotypes and specify the corresponding parameters, 2)to visualize and download the phenotype similarities. Besides, users can submit a list of phenotypes to predict the genes or diseases associated with the given phenotype set.

### User inputs

**Fig. 1:**
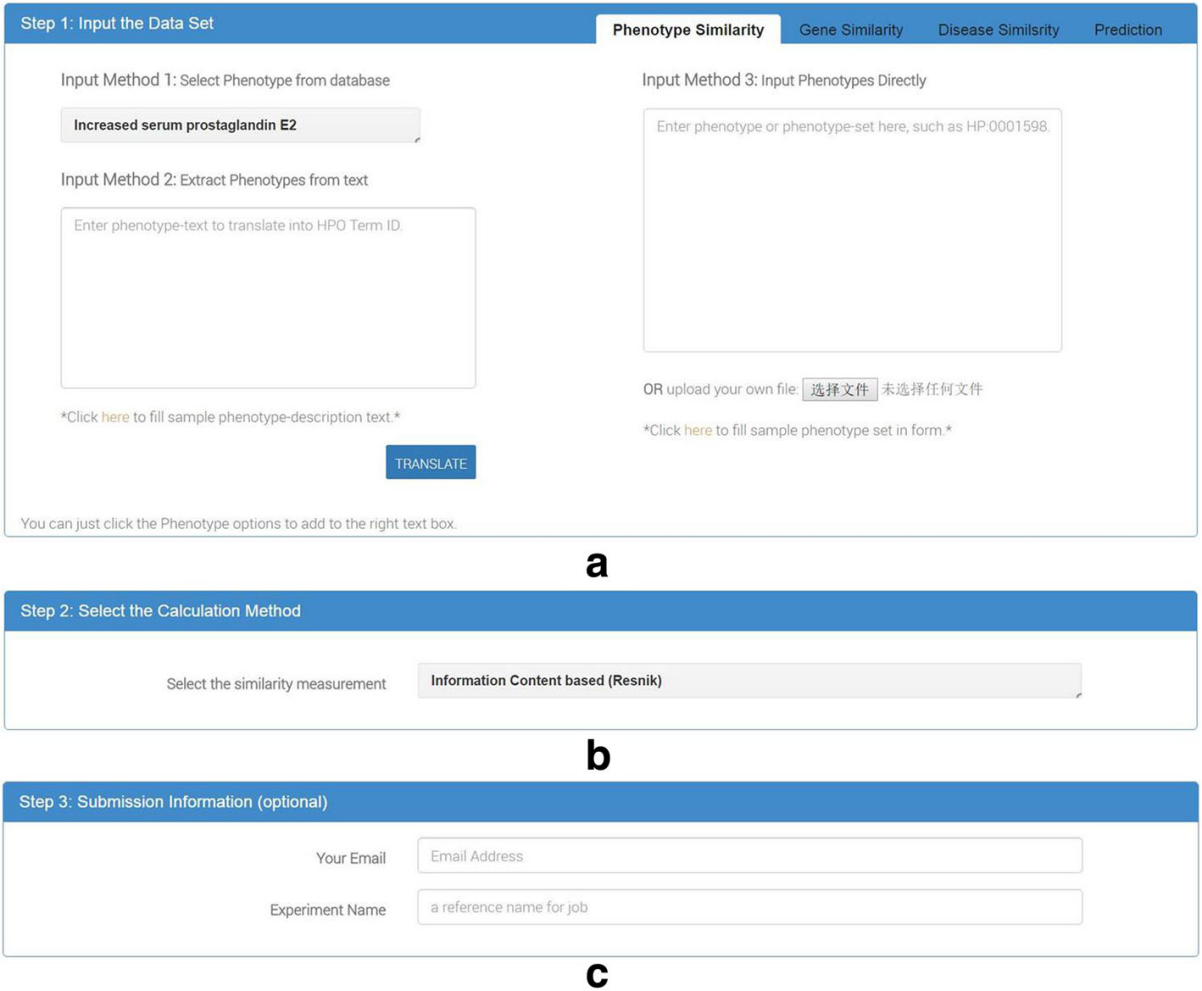
The main input webpage of PhenoSimWeb. The whole process can be divided into three parts, including: **a**) inputting phenotype, gene, or disease dataset, **b**) choosing phenotype similarity measurement, **c**) typing in experimental user information optionally

The user interface of *PhenoSimWeb* can be divided into three parts: phenotypes input (Fig. 1a), similarity measurement selection (Fig. 1b), and user information input (Fig. 1c).

*PhenoSimWeb* mainly contains three functional modules: (1) given a list of phenotypes, calculate the pairwise similarities among the input phenotypes; (2) given a list of genes or diseases, calculate the pairwise similarities by aggregating the similarities of phenotypes associated to given genes or diseases; (3) given a list of phenotypes, identify the most associated genes or diseases with the given phenotypes based on their HPO-based similarity. The input interface for each functional module is introduced as follows.

#### Input interface for phenotype similarity calculation

*PhenoSimWeb* provides three methods for user to input a phenotype list. User can input text that describes phenotypes, select phenotypes from existing databases, and input a set of phenotypes directly (see Fig. 1a). Allowing text input is important, since patients’ phenotypes are always described in text, such as clinical records. *PhenoSimWeb* uses annotation tool Annotator [39] of the National Center for Biomedical Ontology (NCBO) to convert input text to corresponding HPO terms. For the other two input methods, Only HPO ID and Name are allowed in current version.

#### Input interface for gene (or disease) similarity calculation

**Fig. 2:**
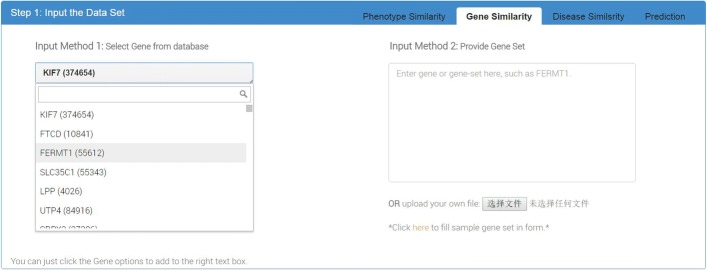
The input webpage of calculating genes similarity. This part provides two types of input, including inputing gene set directly and selecting gene from database

*PhenoSimWeb* provides two methods for user to input a gene (or disease) list. User can select genes or diseases from existing databases, and input a set of genes or diseases directly (see Fig. 2). Currently, *PhenoSimWeb* can only calculate similarities for genes or diseases that annotated by HPO terms, since their similarities are based on the HPO-based phenotype similarities.

#### Input interface for phenotype associated gene or disease prediction

**Fig. 3:**
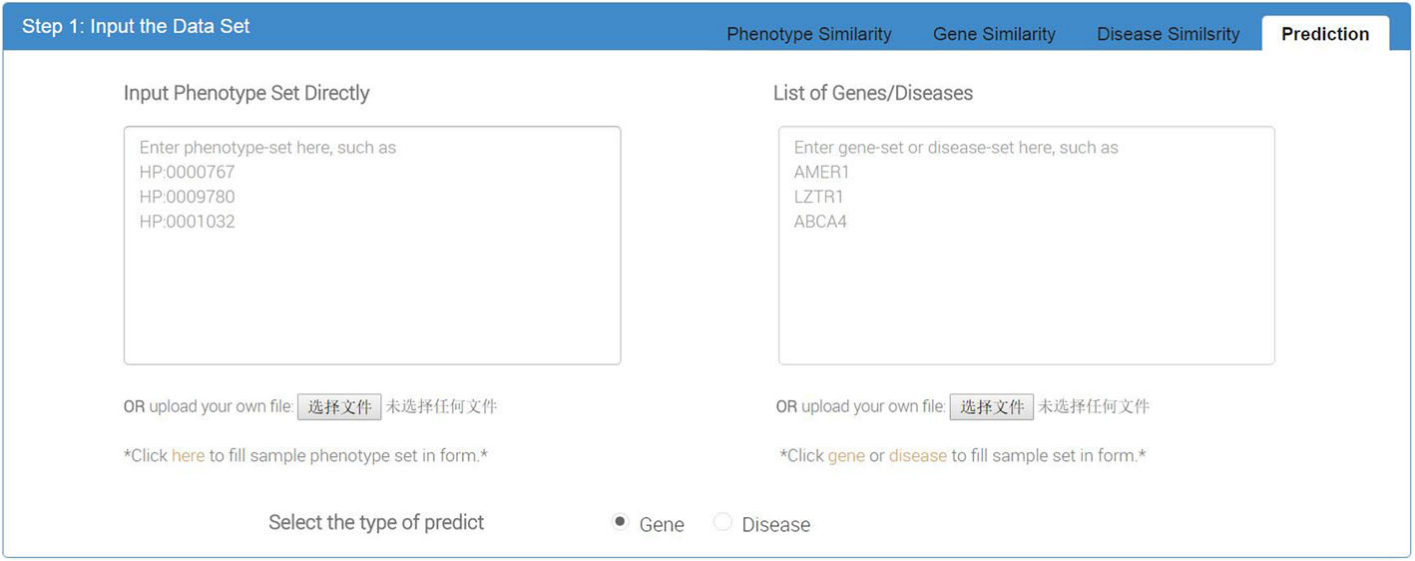
The input webpage of predicting similar genes or diseases. Users input phenotype set in the left text box, gene or disease set in the right text box and select the type of predict

In this part (Fig. 3), users can input phenotype set in the left text box and select the type of target to be predicted, such as gene or disease. Users can also provide a list of target genes or diseases in the right text box to check whether these genes or diseases are associated with the input phenotype set. If the user do not provide a specific gene or disease set, *PhenoSimWeb* would compare the phenotype set with all the genes or diseases involved in HPO.

After the data input step, users can select a semantic similarity measurement for phenotype similarity calculation. A new proposed measurement named *PhenoSim* and other four widely-used similarity measurements are available to choose. The detailed descriptions of these measurements are in the following subsection.

In the last step, users can input email address and the experimental user name optionally. And if users do it, the application will send a notification to the specified mailbox when all the job has been done. And the application will validate it for error checking if all the input information is submitted. The validation process mainly checks the format of input phenotypes, phenotype lists, phenotype texts, genes, genes lists, diseases, diseases lists and all the user specified parameters. And if the input exists any errors, users would be notified immediately. After the validation process, the application will calculate the similarity using specified measurement, which users chose in step two, among phenotypes, genes or diseases, and visualize the phenotype associated network.

All the submitted jobs are executed by a job scheduler on the back-end server of *PhenoSimWeb*. Once all the jobs are finished, a notification email will be sent to the specified mailbox, if users typed in email address in step three. Also, the web will jump to the experimental result’s webpage, if the user unclose the submission webpage and keep it on.

**Fig. 4:**
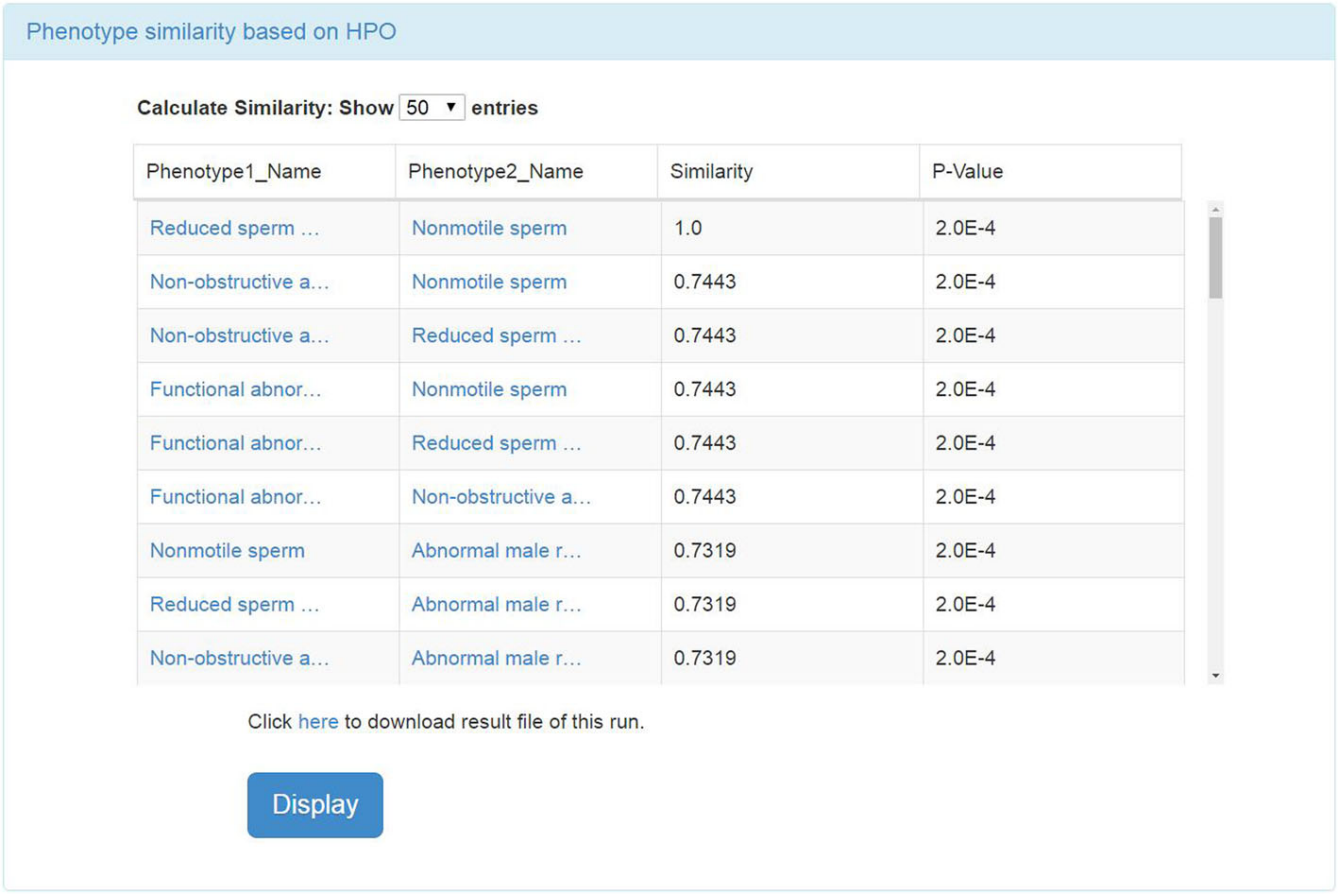
The calculation results of the phenotype list. And *PhenoSim* calculated the corresponding P-Value in addition to the semantic similarity

The experimental result webpage displays the detailed similarity calculation results and corresponding p-values (Fig. 4). The other detailed information in the calculation precess, such as the calculation method, is also displayed on the result webpage. Besides, users can download the experimental result and corresponding information by the links on the webpage.

### Visualization interface

**Fig. 5:**
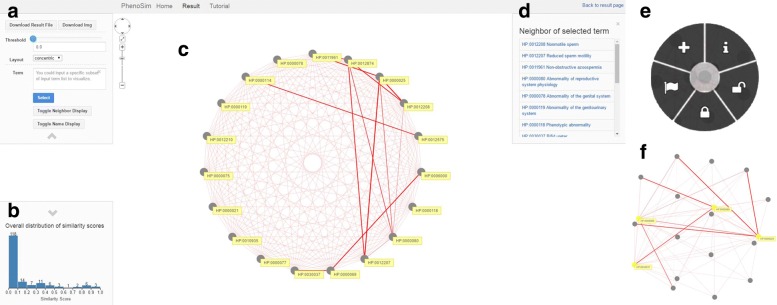
The visualization interface of *PhenoSimWeb* to explore phenotype, gene or disease functional similarities based on HPO. Panel **c** shows the term association network, and among the association network a node indicates a term (phenotype, gene or disease) and an edge between any two interconnected terms indicates that the edge similarity score is greater than the edge similarity threshold, which users input in panel **a**. Panel **b** shows the overall distribution of similarity scores for all the input term pairs, users can regulate the edge similarity threshold in panel **a** by this distribution intuitively. The neighbors of the recently chosen terms are shown in panel **d**. Users can add, info, lock, unlock and flag a term in the node operation panel **e**. Besides, panel **f** show user’s selected subnetwork

*PhenoSimWeb* supplies an intuitional and functional visual webpage to display the similarity results. The visualization interface of *PhenoSimWeb* (see Fig. 5) displays the resulting phenotype association network, and gene or disease association network based on corresponding phenotype similarities in the visualization webpage, in which a node represents a term, such as phenotype or gene or disease, and an edge between any two interconnected terms indicates that the edge similarity score is greater than the edge similarity threshold, which users input in Fig. 5a. Users can implement interactive browsing of the visual interface using the mouse conveniently (Fig. 5c). Besides, users can also activate the node operation panel by long-right clicking a node (see panel in Fig. 5e). Using the node oprtation panel, users can execute multiple node operations, such as: insert current term into selected list on panel A, display term info in top-right panel D, insert term into locked list, delete current term from locked list and set current node’s background color into green.

Users can drag the threshold bar or type in a specific value directly to adjust the edge similarity threshold, and the network will change simultaneously (see Fig. 5a). *PhenoSimWeb* also provides several different graph layouts for graph visualization (see Table 1). Figure 5b shows the overall distribution of similarity scores for all the input term pairs, users can regulate the edge similarity threshold in panel A by this distribution intuitively. The resulting term association network is browsed in the network displaying panel (see Fig. 5c). Besides, users can specify a term group in panel A or node operation panel to select subnetworks (see Fig. 5f). And the term information panel (Fig. 5d) displays the neighbors of current selected term. By clicking a term ID or name on the information panel, users can get more comprehensive information about this term from website (http://compbio.charite.de).

**Table 1:** The layouts that are supported in the visualizing interface. PhenoSimWeb supports six types of layouts in total

Name	Description
Concentric	This layout displays the association network in a concentric circle.
Breadthfirst	This layout displays the association network in a hierarchical structure.
Circle	This layout displays the association networks in a circle. And the circle layout helps users find out high degree nodes or low degree nodes intuitively.
Cose	Cose is an abbreviation for Compound Spring Embedder. This layout lays out compound graphs using a force-directed simulation.
Cola	This layout displays the association network using a force-directed physics simulation.
Grid	This layout displays the association network in a well-spaced grid.

### An illustrative example of using PhenoSimWeb

In this section, we take the sample list of phenotypes in the website as input to demonstrate how to use *PhenoSimWeb* this web application to calculate the pairwise similairty for a set of phenotypes. We select the “PhenoSim” as the HPO similariy measurement in Fig. 1b. And the parameters in Fig. 1c are optional, user can type in an email address and leave the corresponding user name or not. In the end, we click the “submit” button to submit the job.

**Fig. 6:**
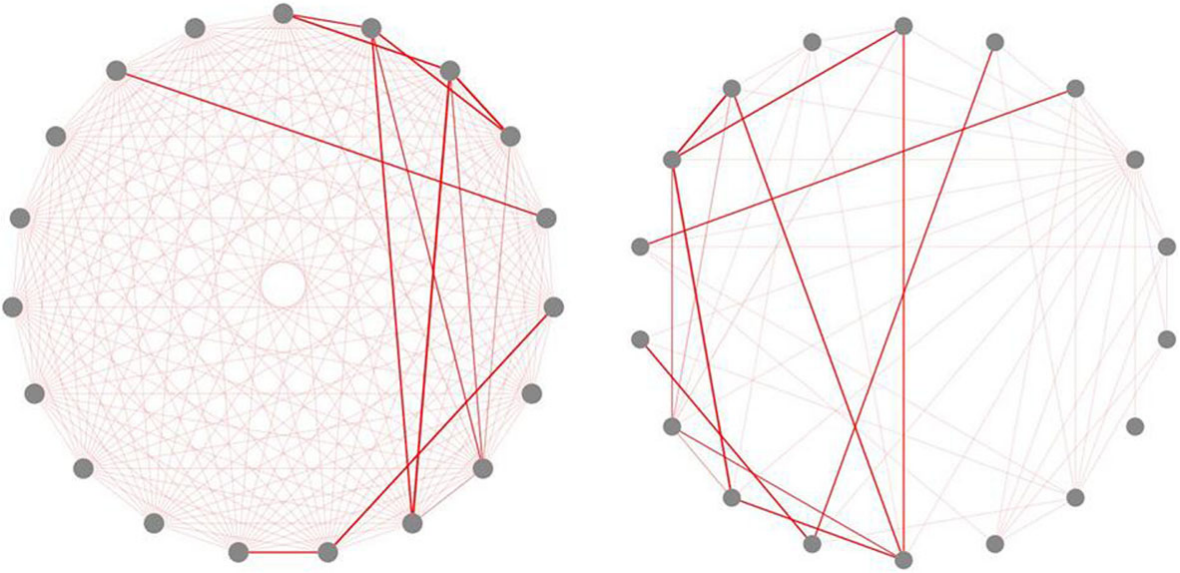
The comparison diagram of two contrasting phenotype association network with different phenotype-to-phenotype similarity thresholds. The edge threshold of left one is 0 and right is 0.1

**Fig. 7:**
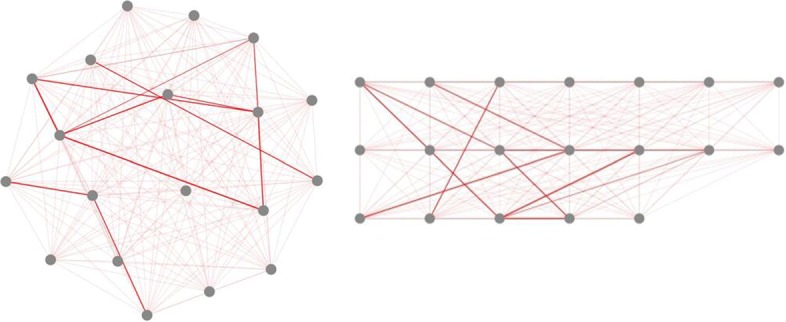
The comparison diagram of visualizing phenotype association network with two different graph layouts. The type of cola and grid are used in the left and right figure respectively

Once all the back-end programs are finished, the calculation results will be displayed on the website (Fig. 4). Users can also download the calculation results by clicking the “Click here to download result file of this run”. Besides, users can click “Display” button to view the graphical visualization of corresponding experimental results (see Fig. 5). By adjusting the phenotype-to-phenotype similarity threshold in panel A, we could obtain two contrasting phenotype association networks (see Fig. 6). In addition, we can also display the association network with different layouts, which are interpreted in Table 1, i.e.,cola and grid, by selecting graph layouts in panel A (see Fig. 7).

**Fig. 8:**
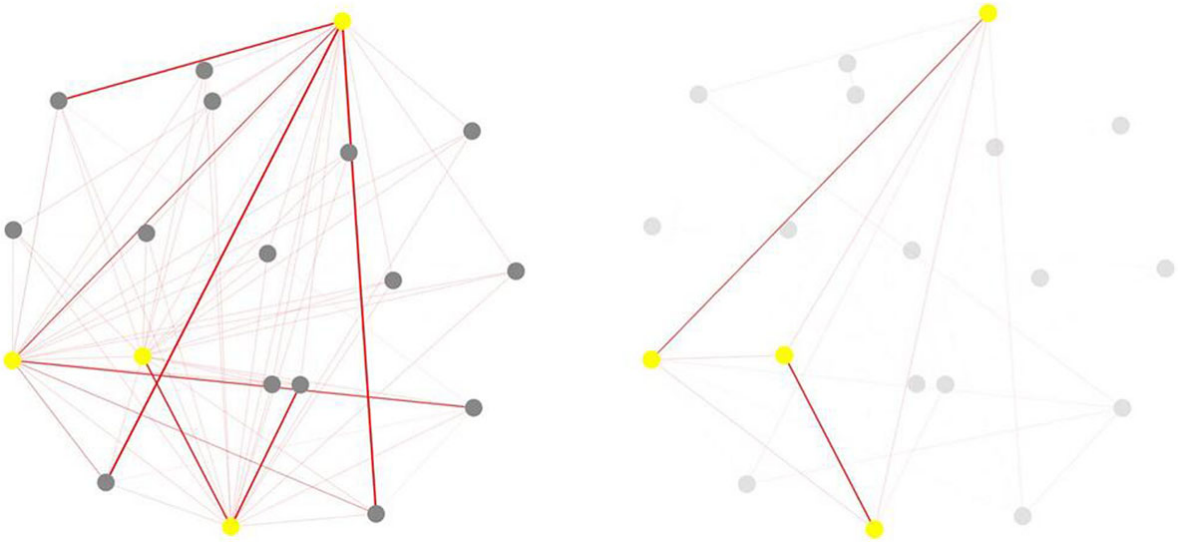
The comparison diagram of constructing subnetworks by selecting interested phenotypes. The right one displays that four interested phenotypes (HP:0000080, HP:0000069, HP:0030037 and HP:0000025) are chosen. The left one displays all the chosen nodes and their direct connected neighbors

In addition to the above functions, we can also choose several phenotypes (i.e., HP:0000080, HP:0000069, HP:0030037 and HP:0000025) as the interested phenotypes and append them into the blank box in panel A using node operation panel. Then the corresponding subnetwork, which contains the selected phenotypes, are highlighted (the right figure in Fig. 8). Besides, users can also add all the neighbors of interested phenotypes into the highlighted network by clicking “Toggle Neighbor Display” in panel A (the left figure in Fig. 8). Furthermore, users could see the detail of each phenotype by clicking nodes among the network in panel C, and the detailed information of the chosen phenotype will be shown in Fig. 5d.

### Implemented similarity measurements

*PhenoSimWeb* provides five HPO-based semantic similarity measures for all the users. We will briefly introduce these five measurements in the following part.

#### 1) *PhenoSim*

In briefly, *PhenoSim* is a path-constrained Information Content-based method for phenotype semantic similarity measurement and includes a noise reduction component to model the noisy patient phenotype data [36]. The whole process of *PhenoSim* contains three steps: First, it constructs a phenotype network *N* using phenotype ontologies and gene-phenotype associations. Second, given a set of clinical phenotypes of a patient, it filters noises based on *N* using PageRank. Finally, it computes the phenotype similarities with a novel path-constrained Information Content-based method.

Compared with other existing approaches, *PhenoSim* effectively improves the performance of the phenotype similarity measurement, and enhances the accuracy of phenotype-based causative gene and disease prediction.

#### 2) Information content based (Resnik)

Resnik et al. [40] proposed a method to calcualte Ontology-based semantic similarity between any two phenotype ontologies, by integrating Information Content (IC) with the Ontology structure. The information content of any term represents the specificity of the term. The terms at a lower level of Ontology tend to have higher IC, and vise verse. In addition, the IC of two phenotype terms is the lowest common ancestor of these two terms in the ontology structure. Given ontology term *t*, and the corresponding information content of *t* could be defined as *IC*(*t*)=−*log*(|*D*_*t*_|/|*D*|), where *D*_*t*_ and *D* are sets of diseases annotated to *t* and the root term. Mathematically, given any two ontology terms *t*_*a*_ and *t*_*b*_, let *t*_*MICA*_ represents their Most Informative Common Ancestor (MICA), the semantic similarity of *t*_*a*_ and *t*_*b*_ is calculated as follows:

1 $$ {Sim}_{Resnik}(t_{a}, t_{b}) = IC(t_{MICA}) = -log\frac{|D_{t_{MICA}}|}{|D|}  $$

where $D_{t_{MICA}}$ and *D* represent the set of annotations of *t*_*MICA*_ and the set of all the annotations involved in the Ontology, respectively.

#### 3) Enhanced information content based (Lin)

Lin et al. [41] considered the Information Content (IC) of two terms *t*_*a*_ and *t*_*b*_ besides the IC of their most informative common ancestor, comparing with the Resnik measure. And the equation of calculating the Ontology term similarity is defined as:

2 $$ {Sim}_{Lin}(t_{a}, t_{b}) = \frac{2 \times IC(t_{MICA})}{IC(t_{a})+IC(t_{b})}  $$

#### 4) Normalized information content based (Schlicker)

Schlicker et al. [35] normalized the Information Content based measure (Resnik) and utilized a weighting function to regulate the overall score:

3 $$ {Sim}_{Schlicker}(t_{a}, t_{b}) = \frac{2 \times IC(t_{MICA})}{IC(t_{a})+IC(t_{b})} \times \left(1 - \frac{|D_{t_{MICA}}|}{|D|}\right)  $$

#### 5) Jiang-Conrath Measure (JC)

Comparing the Resnik measure, Jiang-Conrath [34] considered the information content of term *t*_*a*_ and *t*_*b*_ and the distance between the most public common ancestor besides the information content of *t*_*a*_ and *t*_*b*_. And Jiang-Conrath calculates semantic similarity as:

4 $$ {Sim}_{JC}(t_{a}, t_{b}) = \frac{1}{dist(t_{a},t_{b})+1}  $$

5 $$ dist(t_{a},t_{b}) = IC(t_{a}) + IC(t_{b}) - 2 \times IC(t_{MICA})  $$

## Conclusions

The Human Phenotype Ontology (HPO) is a kind of widely used bioinformatics resources. Recently, various approaches and online or offline tools have been developed to calculate phenotype semantic similarities based on HPO. In this paper, we developed and presented a novel and functional web application, named *PhenoSimWeb*, which allows researchers to compute phenotype similarity with five different measurements conveniently and visualize the resulting phenotype association networks with an easy-to-use and powerful web visualization interface. *PhenoSimWeb* allows text that describes phenotype features as input. *PhenoSimWeb* includes three main functional modules: calculate the pairwise similarities for the input phenotypes; calculate the gene or disease similarities by aggregating the similarities of phenotypes corresponding to the given genes or diseases; identify the most associated genes or diseases with the given phenotype set. In summary, *PhenoSimWeb* is a novel and convenient web application for users to calculate and visualize HPO-based phenotype similarities.
